# Androgen receptor reprogramming demarcates prognostic, context-dependent gene sets in primary and metastatic prostate cancer

**DOI:** 10.1186/s13148-022-01278-8

**Published:** 2022-05-04

**Authors:** Tesa Severson, Xintao Qiu, Mohammed Alshalalfa, Martin Sjöström, David Quigley, Andries Bergman, Henry Long, Felix Feng, Matthew L. Freedman, Wilbert Zwart, Mark M. Pomerantz

**Affiliations:** 1 Division of Oncogenomics, Oncode Institute, Netherlands Cancer Institute, Amsterdam, The Netherlands; 2 Department of Medical Oncology, Dana-Farber Cancer Institute, Boston, MA USA; 3 Department of Radiation Oncology, UCSF Helen Diller Family Comprehensive Cancer Center, San Francisco, CA USA; 4 Division of Oncology, Department of Clinical Sciences Lund, Faculty of Medicine, Lund University, Lund, Sweden; 5 Laboratory of Chemical Biology and Institute for Complex Molecular Systems, Department of Biomedical Engineering, Eindhoven University of Technology, Eindhoven, The Netherlands

**Keywords:** Prostate cancer, Androgen receptor, Epigenome, Transcriptome

## Abstract

The androgen receptor (AR) is a prostate master transcription factor. It binds to genetic enhancers, where it regulates gene activity and plays a fundamental role in prostate pathophysiology. Previous work has demonstrated that AR-DNA binding is systematically and consistently reprogrammed during prostate tumorigenesis and disease progression. We charted these reprogrammed AR sites and identified genes proximal to them. We were able to devise gene lists based on AR status within specific histological contexts: normal prostate epithelium, primary prostate tumor, and metastatic prostate cancer. We evaluated expression of the genes in these gene sets in subjects from two distinct clinical cohorts—men treated with surgery for localized prostate cancer and men with metastatic prostate cancer. Among men with localized prostate cancer, expression of genes proximal to AR sites lost in the transition from normal prostate to prostate tumor was associated with clinical outcome. Among men with metastatic disease, expression of genes proximal to AR sites gained in metastatic tumors was associated with clinical outcome. These results are consistent with the notion that AR is fundamental to both maintaining differentiation in normal prostate tissue and driving de-differentiation in advanced prostate cancer. More broadly, the study demonstrates the power of incorporating context-dependent epigenetic data into genetic analyses.

**Supplementary Information:**

The online version contains supplementary material available at 10.1186/s13148-022-01278-8.

## Introduction

Landmark studies have demonstrated that prostate tumors harbor a relatively low mutational burden compared to other tumor types [1]. Sequencing of localized, early-stage prostate cancer has demonstrated very few recurrent mutations [1, 2]. In advanced late-stage disease, genetic sequencing has revealed few recurrent mutations across cases, with a long tail of low-prevalence mutational drivers [3, 4]. By contrast, the *epigenomic* landscape of prostate cancer appears to undergo highly recurrent alterations—in particular, alterations to chromatin binding patterns of transcriptional regulators such as the androgen receptor (AR) [5]. The AR cistrome—the genome-wide set of AR-DNA binding sites—is consistently reprogrammed during state transitions at thousands of sites [6]. We previously reported the systematic reprogramming of the AR cistrome during both transformation from normal prostate epithelium to prostate cancer [6] and during progression from localized prostate tumors to metastatic castration-resistant prostate cancer (mCRPC) [5]. In each state-to-state transition, AR relocates to alter the activity of enhancers, intergenic elements that regulate expression of distal genes. The shifts in the AR cistrome during these state-to-state changes are distinct from one another, resulting in thousands of state-specific AR binding sites correlated with the expression of hundreds of genes.

Given the consistency of cistromic changes during tumorigenesis and again during metastatic progression, we hypothesized that genes governed by these enhancers carry state-specific, clinically relevant information. Simply, we posited that these state-specific gene sets are prognostic. Clinical context is a crucial aspect of this hypothesis. We reasoned that a gene set defined by a state-specific AR cistrome will influence outcome only within that distinct clinical state. Conversely, a gene set defined by the AR cistrome in one state will not be associated with outcomes at different stages of disease.

We compiled gene sets at the reprogrammed AR sites for two state transitions: tumorigenesis (healthy tissue to primary localized tumor) and metastasis (primary localized tumor to distant metastatic tumor). We then determined associations between these gene sets and clinical outcomes of treatment in two separate prostate cancer cohorts: time to metastasis in patients undergoing radical prostatectomy for localized disease and overall survival (OS) of patients from the time of diagnosis of mCRPC.

## Methods

### Generation of gene sets

To identify gene sets associated with AR binding sites present in normal prostate epithelium and lost in localized prostate tumors and gene sets associated with AR binding sites absent in normal prostate epithelium and gained in tumor, we first catalogued all differentially expressed genes in a large publicly available cohort (TCGA-PRAD) between primary prostate tumors (*n* = 497) and normal prostate epithelium (*n* = 52) [1]. Differential expression was identified using DESeq2 v1.22.2 in R v3.5.0. We next sought the subset of significantly differentially expressed genes that reside proximal (≤ 50 kb) from tissue-specific AR binding sites. This distance was selected because it captures a large proportion of possible enhancer/gene pairings while maintaining modestly sized gene sets for analysis [7]. AR binding sites unique to normal prostate epithelium vs. localized prostate tumor (and the converse) were defined as previously described [6]. If the closest gene was differentially expressed in the appropriate direction (i.e., up-regulated in tumor for AR binding sites unique to prostate tumor and down-regulated in AR sites unique to normal epithelium), it was selected, using BedTools v2.26.0. In this manner, we leveraged the initial information about gene expression to query AR binding in an additional dataset in order to better define features which are not specific to a dataset but rather the state transition. The resulting gene sets were labeled “lost in tumor” (LiT) for genes down-regulated and proximal to AR sites unique to normal epithelium; and “gained in tumor” (GiT) for genes up-regulated in tumor and proximal to AR sites unique to prostate tumor. We similarly defined “lost in metastases” (LiM) and “gained in metastases” (GiM) genes in the same manner using the microarray appropriate R package limma (v3.38.3) to determine differentially expressed genes from a cohort comprised of primary tumor (*n* = 131) and metastatic tumor (*n* = 19) [8] specimens. Localized tumor-specific and metastatic tumor-specific AR sites [5] were then used in construction of the LiM and GiM gene sets in the manner described above (Additional file 2: Figure S2 and Additional file 4: Table S1). Significant enrichment of gene sets in the KEGG pathways database was examined and visualized using ClusterProfiler and DOSE R packages in R [9, 10] and the MSigDB database [11, 12].

### Testing prognostic capacity of gene lists

Using R, we grouped patients in each clinical cohort into two using the quantiles of the average gene exp (cutoff at 0.75 quantile) for each gene list. Next, we used the survival package in R to determine the statistical significance of association with survival in each cohort (Wald statistic). To plot patient stratification, we used Kaplan–Meier curves. Clinical details of each cohort are previously described [4, 13] and were independent of the cohorts used to derive gene sets in order to avoid overfitting of the data. Among subjects undergoing radical prostatectomy, “high-risk” was defined as any of preoperative PSA > 20 ng/mL, pathological Gleason score ≥ 8, seminal vesicle invasion, or Gleason/PSA/pathologic stage score ≥ 10 [13, 14]. Both cohorts were censored at last follow-up.

## Results

**Fig. 1:**
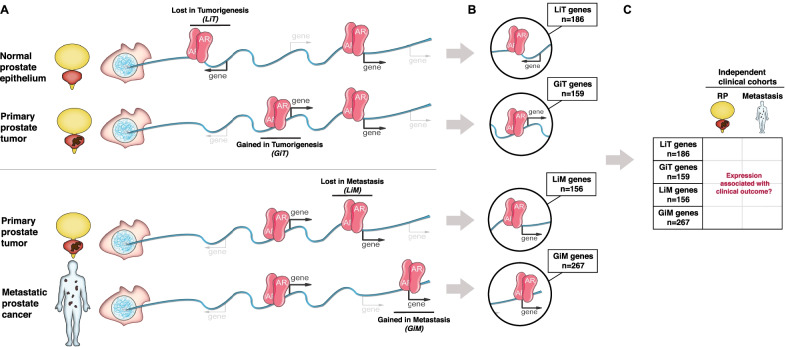
Schematic of the study methodology. **A** Schematic of AR biding sites unique to specific clinical states. Depicted are AR sites specific to healthy prostate epithelium and lost in primary prostate tumors (LiT)—i.e., AR is present in the first DNA strand representing the normal prostate genome and absent in the second strand representing the localized prostate tumor genome; AR sites absent in primary prostate epithelium and gained in primary prostate tumors (GiT); AR sites present in primary prostate tumors and lost in prostate cancer metastases (LiM); and AR sites absent in primary prostate tumors and gained in prostate cancer metastases (GiM). **B** Gene sets were selected based on their differential expression across tumor types and their proximity (≤ 50 kb) to tissue-specific AR sites. The gray arrows from each DNA strand direct to a highlighted region from that strand exemplifying a context-specific AR site (LiT, GiT, LiM, or GiM) and its associated gene. The number of genes identified genome-wide from each category are indicated in the text box linked to the representative genomic regions. **C** Clinical outcome based on expression of the genes within each individual cohort (LiT, GiT, LiM, or GiM along the y-axis) was examined in two independent cohorts: a localized disease cohort (RP, radical prostatectomy) and a metastatic cohort (metastasis) as depicted across the x-axis. In total, eight independent analyses were performed

To identify gene sets of interest, we selected recurrently lost and gained AR binding sites using previously generated epigenetic datasets in human prostate cancer subjects with localized prostate cancer or mCRPC [5, 6]: (i) AR binding sites *lost* in the transition from normal prostate epithelium to localized prostate tumor—lost in tumorigenesis (LiT); (ii) AR binding sites *gained* in the transition from normal prostate epithelium to localized prostate tumor—gained in tumorigenesis (GiT); (iii) AR binding sites present in localized tumor but *lost* in mCRPC tumors—lost in metastasis (LiM); and (iv) AR binding sites uniquely *gained* in mCRPC tumors—gained in metastasis (GiM) (Fig. 1A). Of note, the mCRPC subjects had not yet received AR pathway inhibitors. We identified all genes located within 50 kilobases (kb) from each of these four AR binding site categories using gene expression data from large well-known patient cohorts of normal versus primary tumor[1] and primary tumor versus metastasis [8] by selecting genes whose expression tracked with original AR binding status (Additional file 1: Fig. S1A/B).

Specifically, we used the mRNA expression data set from The Cancer Genome Atlas (TCGA) to determine differential gene expression between normal prostate and localized tumors [1] and used the mRNA expression set generated by Taylor et al., to determine differential gene expression between localized prostate cancer and metastatic disease [8]. At LiT AR sites, we selected genes that were up-regulated in normal prostate epithelium relative to local tumor (*n* = 186); at GiT AR sites, we selected genes up-regulated in localized prostate tumor relative to normal epithelium (*n* = 159); at LiM AR sites, we selected genes up-regulated in local tumor relative to prostate metastases (*n* = 156); and at GiM AR sites, we selected genes up-regulated in prostate metastases relative to local tumor (*n* = 267) (Fig. 1B).

The ability of the four gene lists to predict patient outcome was then examined using patient gene expression and survival data from two independent sources: (i) 780 high-risk prostate cancer subjects with radical prostatectomy (HRRP) material [13] and (ii) 96 mCRPC patients with biopsy material from a metastatic site [4] (Fig. 1C). These clinical cohorts represent two distinct stages in the natural history of prostate cancer—primary prostate cancers and castration-resistant metastatic disease. For the HRRP cohort, clinical outcome was determined by assessing metastasis-free survival [13]. For the metastatic cohort, clinical outcome was determined by assessing overall survival from the time of mCRPC diagnosis [4].

There were eight tests in total—the four gene sets (LiT, GiT, LiM, and GiM) across the two clinical cohorts (HRRP and mCRPC). For each gene list, we dichotomized patients in the respective clinical cohorts into two groups defined by the average gene expression of the gene list in the cohort (cutoff at 0.75 quantile average gene expression (above/below)). We then determined whether these gene list expression-based groupings were associated with clinical outcome using the Kaplan–Meier analysis and log-rank test.

**Fig. 2:**
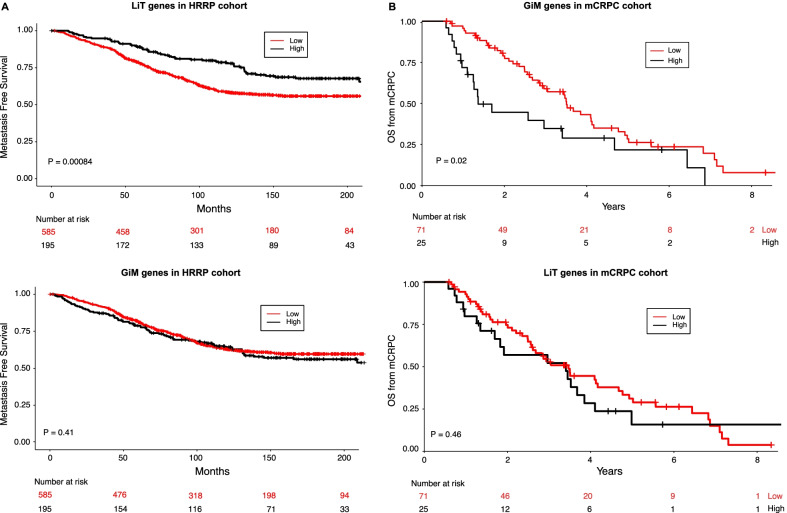
Survival analysis of LiT and GiM gene sets in different cohorts. **A** Kaplan–Meier curves of the LiT (top) and GiM (bottom) genes in the primary tumor (HRRP) cohort. **B** Kaplan–Meier curves of GiM (top) and LiT (bottom) genes in the metastatic (mCRPC) cohort

Expression levels of genes proximal to AR sites unique to normal prostate epithelium (LiT) were significantly associated with metastasis-free survival in the HRRP cohort (*p* = 8.4 × 10^–4^, Fig. 2A). Conversely, the GiT genes did not associate with outcome in this cohort (*p* = 0.08) (Additional file 2: Figure S2), nor did the two gene sets differentially enriched in the metastatic state transition (GiM genes *p* = 0.41; LiM genes *p* = 0.24) (Fig. 2A, Additional file 2: Figure S2). These results suggest that genes regulated by AR binding sites specific to normal, mature prostate epithelial cells are associated with outcome in primary prostate cancer.

Next, for all four gene sets we assessed associations with outcome in the metastatic setting. Here, genes proximal to mCRPC-specific AR binding sites (GiM genes) were associated with overall survival in the metastatic cohort (*p* = 0.02; Fig. 2B), while none of the other gene sets were associated with survival in this setting (LiT genes *p* = 0.46, GiT genes *p* = 0.15; LiM *p* = 0.31; Fig. 2 and Additional file 2: Figure S2). These results indicate that highly expressed genes proximal to mCRPC-specific AR binding sites are significantly associated with more aggressive disease (*i.e*., shorter overall survival). Corroborating this finding, we specifically identified significant enrichment of KEGG cell cycle and DNA replication gene sets in GiM genes (Additional file 3: Figure S3).

## Discussion

We have previously demonstrated highly reproducible alterations in the epigenome in cellular transformation and evolution of the cancer cell [6]. During prostate cancer development and progression, the AR cistrome undergoes systematic changes, accessing certain gene expression programs while abandoning others. These epigenetic changes help shape the phenotype of the cancer cell. We hypothesized that the expression of genes regulated by state-specific AR-bound enhancers would prove clinically informative. Consistent with this hypothesis, we observed that expression of genes proximal to AR sites lost in the transition from normal prostate to prostate tumor was associated with clinical outcomes among men with localized disease. Among men with metastatic disease, expression of genes proximal to AR sites gained in metastatic tumors was associated with clinical outcomes. Our data demonstrate that state-specific epigenetic features can be a useful guide for identifying and refining informative gene sets in specific clinical contexts.

The strongest association between gene expression and clinical outcome was observed at the extremes of the disease’s natural history: genes in normal epithelium prior to tumorigenesis and genes in mCRPC. Specifically, genes at AR sites specific to mature differentiated tissue (LiT genes) and genes at AR sites specific to de-differentiated late-stage cancer (GiM genes) were most prominently associated with outcome. Intriguingly, lower expression of LiT genes and higher expression of GiM genes were associated with deleterious outcomes in their respective clinical settings.

In the localized disease cohort, the results suggest that AR-mediated maintenance of a specific set of genes in mature prostate epithelium discourages de-differentiation and subsequent tumor aggressiveness. This is consistent with previous analyses. Tomlins et al. reported that low-grade localized prostate cancers express AR signature genes more strongly than higher-grade tumors [15]. A meta-analysis of multiple gene expression data sets, validated using TCGA, revealed that levels of androgen-regulated genes correlated inversely with aggressiveness of localized prostate cancer [16]. Our findings suggest that AR sites specific to normal prostate epithelium (*i.e*., lost in tumor) serve to maintain prostate differentiation.

In the metastatic setting, we observe the opposite effect. Increased expression where AR is gained in metastasis is associated with worse outcome. These data are intriguing in light of recent observations in which metastatic prostate cancer cells access and activate fetal prostate developmental programs during progression [5]. It follows that AR activation of these enhancers and subsequent up-regulation of their target genes promote cellular de-differentiation.

We made certain assumptions to compile our gene sets. A central one was that the gene most proximal to a given AR site is the gene regulated by the regulatory element. While it is more likely that a cis-regulatory element will regulate a proximal gene compared to a distal one, this is not always the case. Also, enhancers can regulate several genes within the genome. Emerging technologies that survey chromatin conformation could elucidate enhancer–gene interactions. Another limitation of our study is that we could not functionally annotate each of the thousands of AR sites to more confidently pinpoint gene targets. As large-scale functional analyses become tractable, we anticipate that even more informative gene sets may be assembled.

It is also possible that cistromes other than the AR cistrome or epigenetic alterations other than AR-DNA binding may prove more proficient in identifying useful gene sets. It is important to note that state-specific AR binding coincides with several other intriguing epigenetic features, such as FOXA1 binding, HOXB13 binding, H3K27ac and hypomethylation [5]. Further work will be needed to determine whether the key epigenetic alterations driving the signal observed here are due to AR, another factor, or the interplay among several epigenetic changes. Other weaknesses of our study include the size of the patient populations and a reliance on retrospective clinical data, with limited information regarding specific treatments. In addition, future studies could include patients resistant to AR pathway inhibitors, where AR splice variants may prove informative. It is reflective of the capability of state-specific epigenetic analysis that we were able derive clinically meaningful results based on our broad and imperfect assumptions.

In summary, our findings demonstrate the power of incorporating the epigenome into genomic and transcriptomic analyses. Epigenetics data provided a map for identifying precisely where AR is enacting its program. The resulting gene sets and their associations with patient outcomes reflect, specifically, the key role of AR in shaping the identity of the prostate cancer cell and, more generally, the potential in using the epigenome to create informative gene sets. The results also highlight the importance of clinical context when evaluating the transcriptome.

## Supplementary Information

**Additional file 1. Figure S1**: Selection of genes proximal to state-specific AR sites. A. Flowchart of identification of LiT and GiT genes. B. Flowchart of identification of LiM and GiM genes

**Additional file 2. Figure S2**: Survival analysis of GiT and LiM gene sets in different cohorts. Kaplan–Meier curves of the GiT (top) and LiM genes (bottom) in the primary tumor (HRRP) and metastatic (mCRPC) cohort

**Additional file 3. Figure S3**: MSigDB Canonical Pathways KEGG gene set enrichment. Colorplot indicating the significantly enriched KEGG pathways (MSigDB) identified with the enricher function in the DOSE package. Shown are all pathways which were significant in at least one gene list. Color indicates less (white) to more (red) significant adjusted p values

**Additional file 4**. **Table S1:** List of genes included in each state-specific AR binding gene set and fold-change in gene expression across states.

## Data Availability

Data accessed for the study are publicly available, as described in the referenced manuscripts.

## Abbreviations

AR: Androgen receptor
GiM: Gained in metastases
GiT: Gained in tumor
HRRP: High-risk prostate cancer subjects with radical prostatectomy
kb: Kilobases
LiM: Lost in metastases
LiT: Lost in tumor
mCRPC: Metastatic castration-resistant prostate cancer
OS: Overall survival
TCGA: The Cancer Genome Atlas
