# Supplementary figures and images for: Androgen receptor reprogramming demarcates prognostic, context-dependent gene sets in primary and metastatic prostate cancer
Source: Clin Epigenetics. 2022 May 4;14:60. doi: 10.1186/s13148-022-01278-8 (PMC9069737; doi:10.1186/s13148-022-01278-8)

**Supplementary Figure 1**

**A**

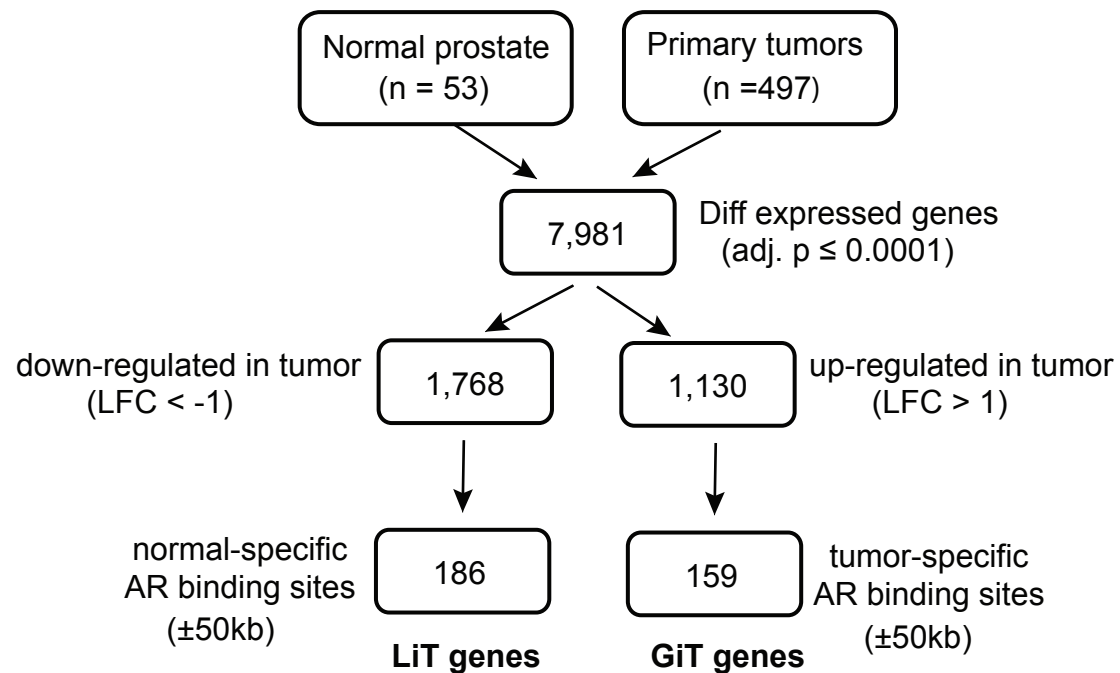

**B**

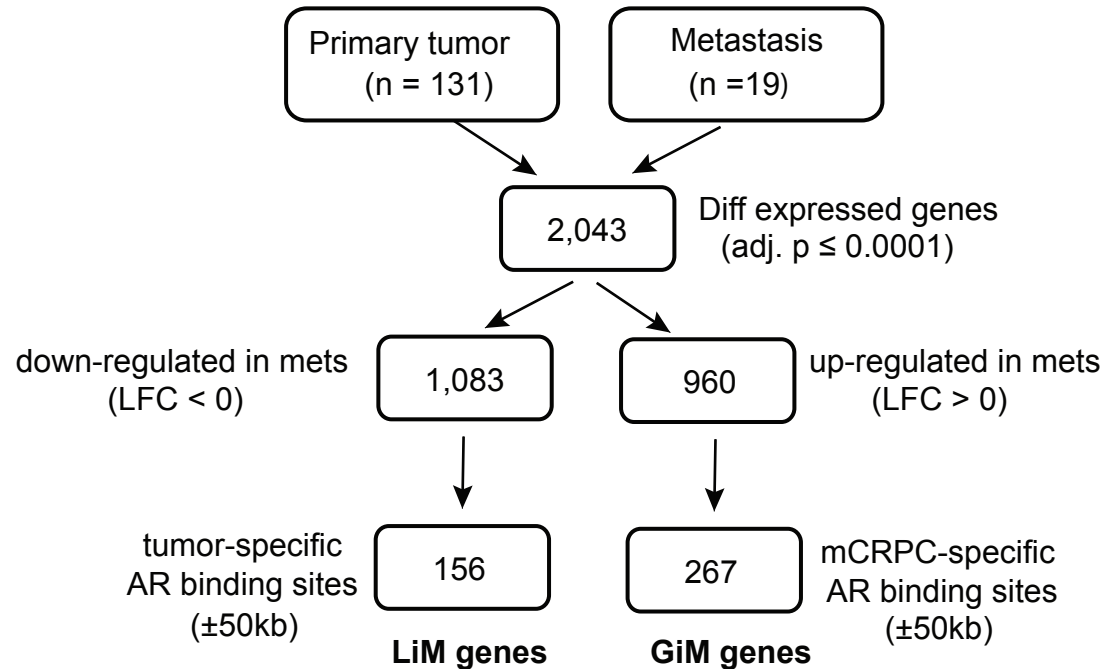

Supplement: Supplementary file 1 — Additional file 1. Figure S1: Selection of genes proximal to state-specific AR sites. A. Flowchart of identification of LiT and GiT genes. B. Flowchart of identification of LiM and GiM genes [file 13148_2022_1278_MOESM1_ESM.pdf]

Supplementary Figure 2

A

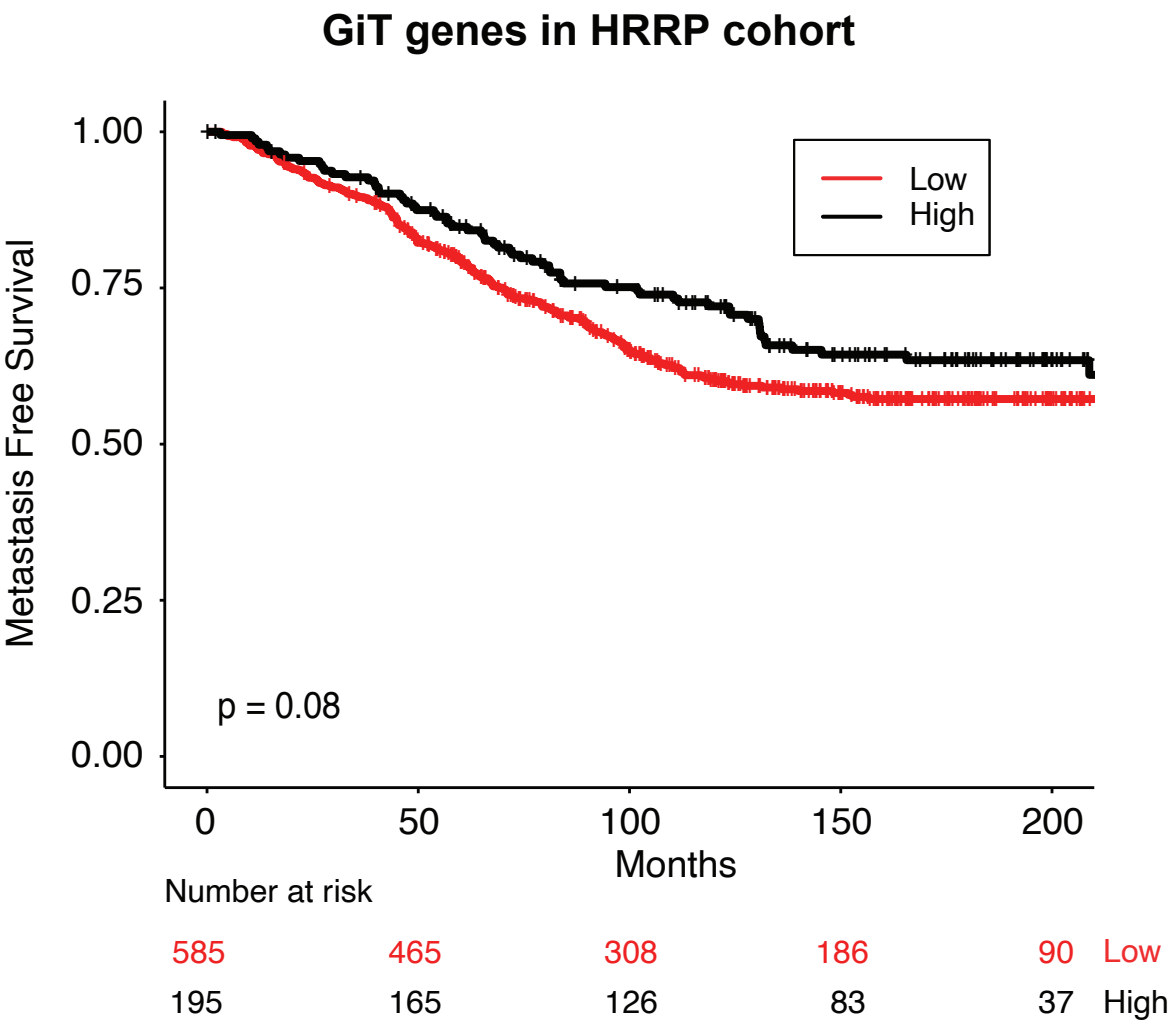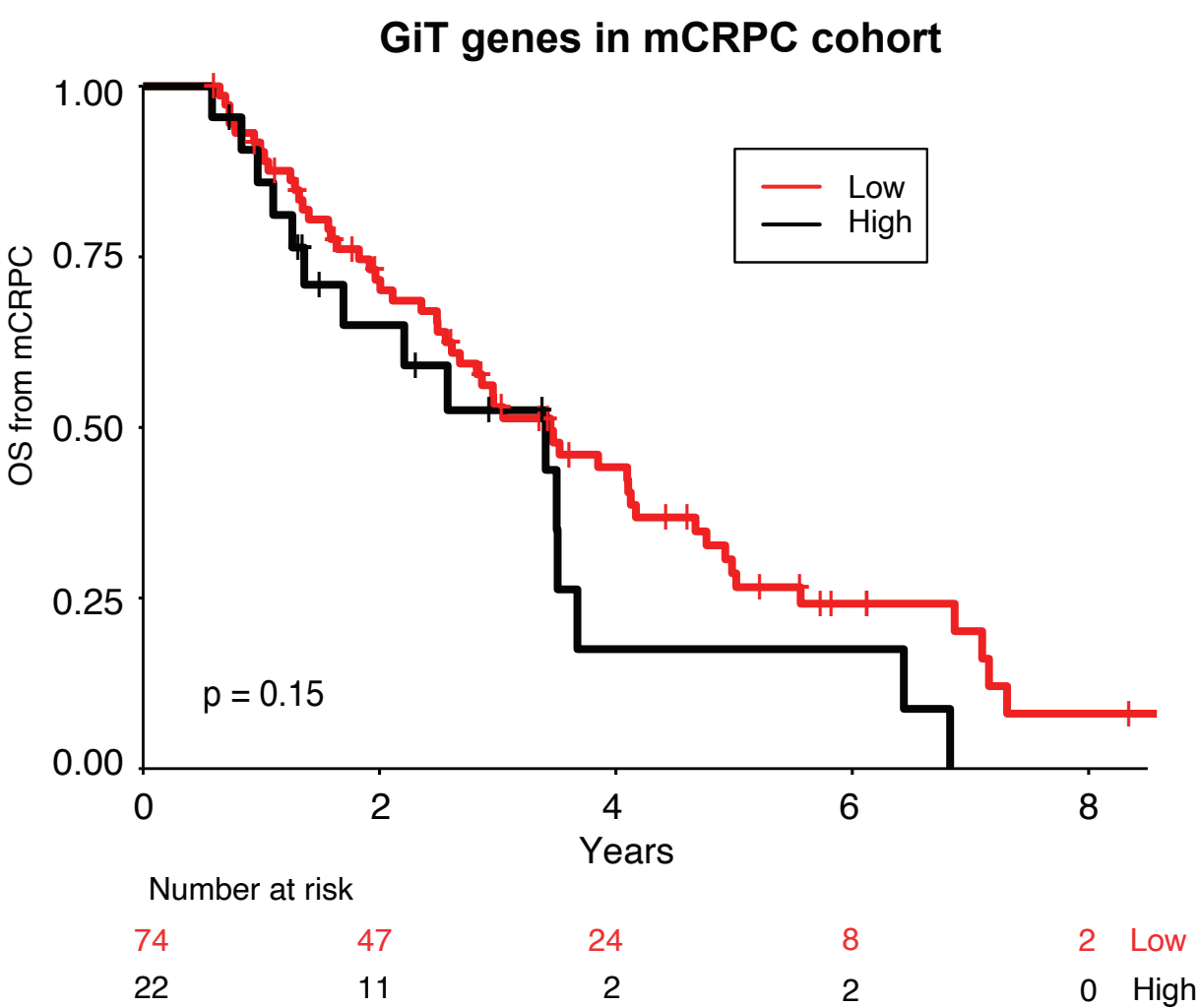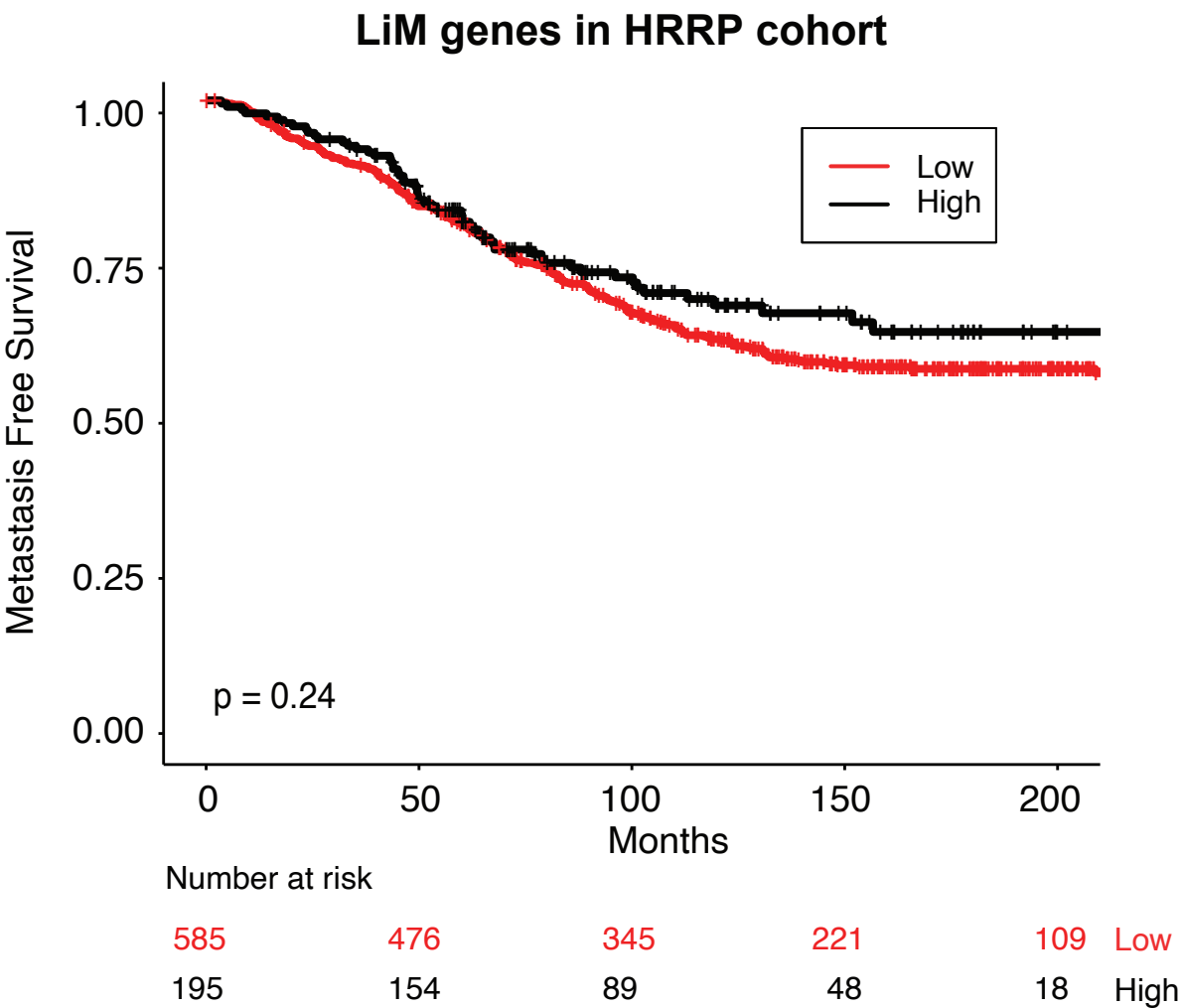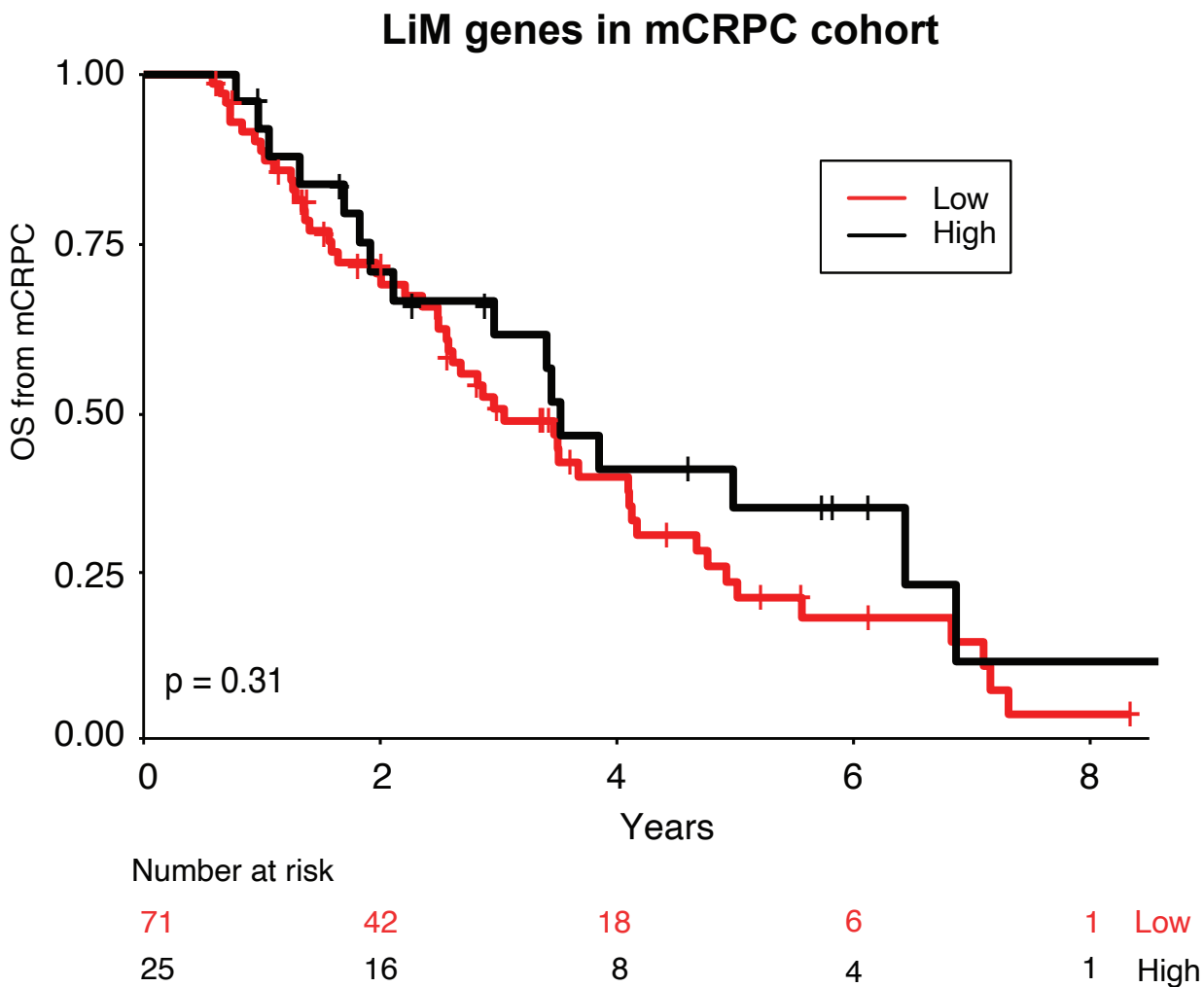

Supplement: Supplementary file 2 — Additional file 2. Figure S2: Survival analysis of GiT and LiM gene sets in different cohorts. Kaplan–Meier curves of the GiT (top) and LiM genes (bottom) in the primary tumor (HRRP) and metastatic (mCRPC) cohort [file 13148_2022_1278_MOESM2_ESM.pdf]

Supplementary Figure 3

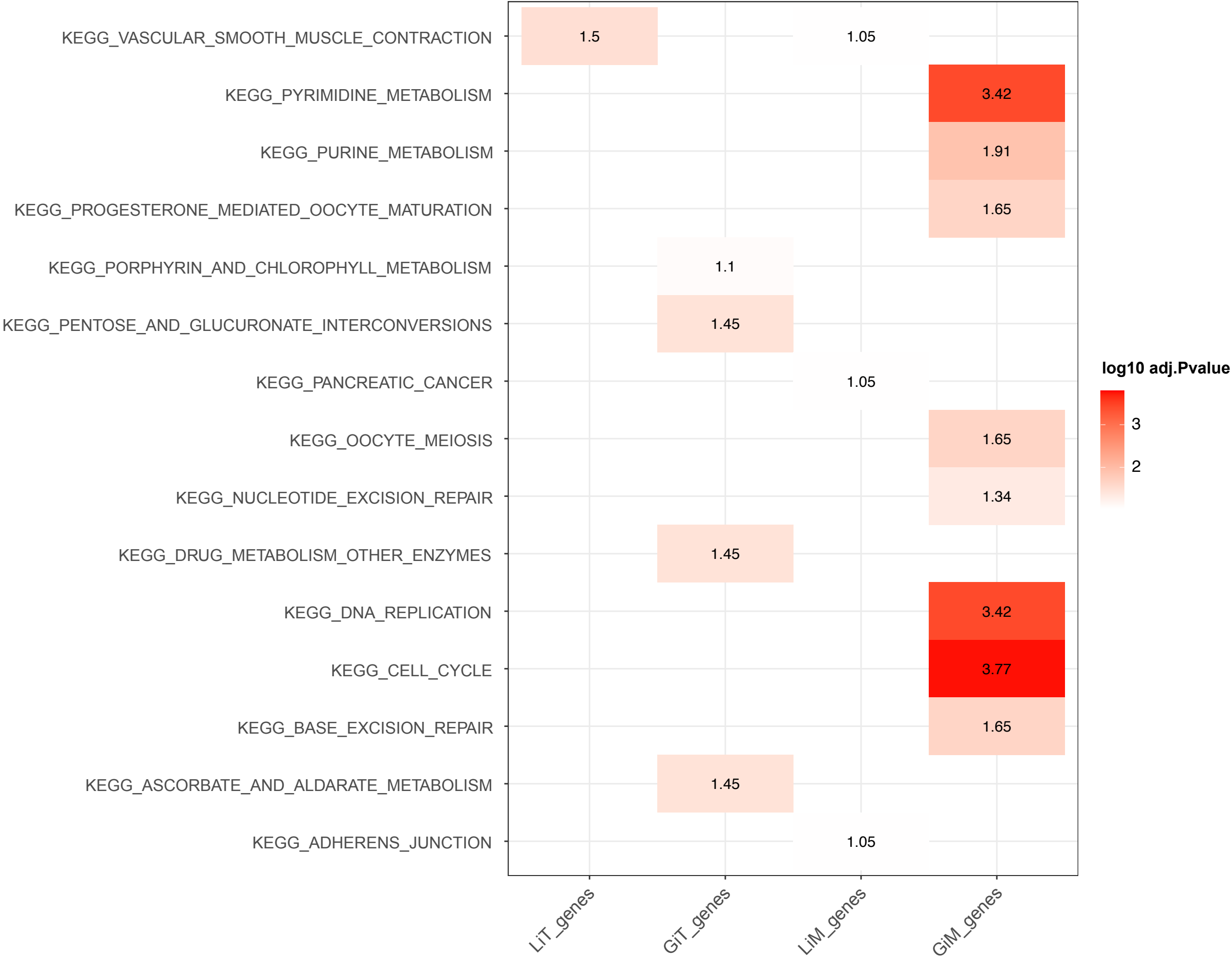

Supplement: Supplementary file 3 — Additional file 3. Figure S3: MSigDB Canonical Pathways KEGG gene set enrichment. Colorplot indicating the significantly enriched KEGG pathways (MSigDB) identified with the enricher function in the DOSE package. Shown are all pathways which were significant in at least one gene list. Color indicates less (white) to more (red) significant adjusted p values [file 13148_2022_1278_MOESM3_ESM.pdf]
